# Wireless Communication Using a Radiation-Type Metasurface

**DOI:** 10.3390/mi16080924

**Published:** 2025-08-11

**Authors:** Jun Chen Ke, Li Wang, Mingzhu Jiang, Qiang Wang

**Affiliations:** 1 School of Optoelectronic Engineering, Guilin University of Electronic Technology, Guilin 541004, China; jcke@guet.edu.cn (J.C.K.); wl163com2025@163.com (L.W.); 2 Postdoctoral Mobile Station for Instrument Science and Technology, School of Electronic Engineering and Automation, Guilin University of Electronic Technology, Guilin 541004, China; 3 Guangxi Key Laboratory of Optoelectronic Information Processing, Guilin University of Electronic Technology, Guilin 541004, China; 4 Guangxi Key Laboratory of Automatic Detecting Technology and Instrument, Guilin University of Electronic Technology, Guilin 541004, China; pearl5607442@126.com

**Keywords:** radiation-type metasurface, wireless communication, microstrip array antenna

## Abstract

The rapid development of metasurfaces offers new possibilities to establish novel wireless communication systems with simplified architectures. However, the current demonstration systems are based on the reflection-type metasurfaces, which suffer from high profiles and integration challenges in practice. Such configurations are also inefficient for handling multiple subcarriers during beam scanning and beam tracking. To address these limitations, a radiation-type metasurface fed by a microstrip array antenna is proposed in this paper, which is used to construct a new-architecture wireless communication system. Compared to the reported metasurface-based communication systems, the proposed design is more flexible for information modulation and transmission, with the system profile significantly reduced. The phase modulation is implemented by changing the transmission phase of metasurface, allowing for baseband signals to be directly imparted to the carrier wave from the feeding source. A real-time signal transmission experiment validates the performance of the proposed metasurface-based communication system.

## 1. Introduction

Metasurfaces, as a kind of patterned interface, have provided unprecedented degree of freedom for wave manipulations in acoustic, electromagnetic (EM), and optical regions [1,2,3]. Local control of the reflection/transmission properties at different positions of the metasurface is accomplished by altering the element shapes, dimensions, and spatial alignment, enabling synthesis of amplitude and phase profiles favored by a number of applications like beamforming, EM stealth, and polarization conversion [4,5,6,7].

However, despite the outstanding properties of the artificial metasurface, the design and optimization of such surface are highly sophisticated, especially when the array size is extremely large. To reduce the design complexity, the concept of coding metasurfaces is proposed [8]. Only finite meta-atoms with $2^{n}$ phase states are considered to constitute the whole surface. The corresponding radiation/scattering characteristics are highly dependent on the coding sequences of the meta-atoms. More importantly, the coding metasurface can be easily extended to a digital, programmable metasurface once the active devices are incorporated in the element design, providing a new path for beam generation and beam scanning in contrast to phased array antennas [9,10,11,12,13,14,15]. Recently, by leveraging the dynamic tailoring of amplitude/phase in different time slices [16,17,18,19], the metasurface-based wireless communication schemes have attracted considerable attentions. Such approach is particularly valued by modern frequency division multiple access communication systems and demonstrates the feasibility of constructing wireless communication systems with simplified architectures [20,21,22].

In previously reported communication systems, only the reflection-type metasurfaces are employed for baseband modulation and signal radiation. Although excellent performances are exhibited by these digital metasurfaces to meet the demands of wireless data transmission, there are still some disadvantages like the large volume and high profile, that pose great challenges to reduce the overall size and integrate the metasurface into the whole system. In terms of the interactions between EM wave and the metasurface, the radiation-type metasurfaces integrate the feeding sources and the metasurfaces, which do not block the incident EM waves, thereby enabling the entire communication system highly integrated and improving spatial utilization.

In this paper, a radiation-type metasurface is designed and exploited to build a wireless communication system, in which a microstrip array antenna source is placed at the back of a transmissive metasurface [23,24]. By establishing the mapping relationship, the baseband data can be easily loaded into the carrier signals by changing the transmission phase of proposed metasurface in real time. The proposed radiation-type metasurface is more flexible to use in practical communication systems than the reflection-type counterpart. A set of measurements are conducted to test the accuracy and reliability of the proposed system. The experimental results confirm the possibility of building a highly integrated communication system for wireless data transmission.

## 2. Theory and Design

In traditional phase-shift keying (PSK) communication systems, the carrier wave typically undergoes several phase reversals. For example, a binary phase shift keying (BPSK) communication scheme needs two phase reversals, specifically 0° and 180°, to represent the binary symbols ‘0’ and ‘1’ during the data transmission. Therefore, the signal modulation can be easily achieved by simply varying the phase of the sinusoid according to the message bits. Specifically, two basic functions, $s_{0}$ and $s_{1}$, can be selected in this modulation scheme as follows:

(1) $$s_{0}=Ae^{j(2\pi ft+\pi )},$$

(2) $$s_{1}=Ae^{j2\pi ft},$$

where *f* is the carrier frequency, and *A* represents the carrier amplitude. The subscripts of s stand for the symbol ‘0’ or ‘1’ respectively. Therefore, at the receiver end, the transmitted information can be easily recovered by detecting the carrier phases in the basic functions after down-conversion to baseband.

Following the same procedure, we aim to replace the baseband modulator by metasurface. Assume that the transmission coefficient of the metasurface $T=T_{m}e^{j\theta }$, where $T_{m}$ and θ are the amplitude and phase of the transmission coefficient. In our design, only the transmission phases are rapidly changed with time, since the amplitude variation will inevitably lead to the material loss within the metasurface, and lessen the propagation distance of EM signals. If the incident electric field is written as $E_{i}\left(t\right)=E_{m}e^{j2\pi ft}$, in which $E_{m}$ stands for the incident electric field amplitude. At normal incidence, the transmitted electric field $E_{t}$ can be given by

(3) $$E_{t}\left(t\right)=E_{m}T_{m}e^{j(2\pi ft+\theta )}.$$

Comparing Equation (3) and Equations (1) and (2) reveals that the baseband information can be directly modulated to the transmitted wave when the transmission phase θ of the metasurface is switched between 0° and 180° depending on the transmitted data, thus realizing the wireless communication.

**Figure 1:**
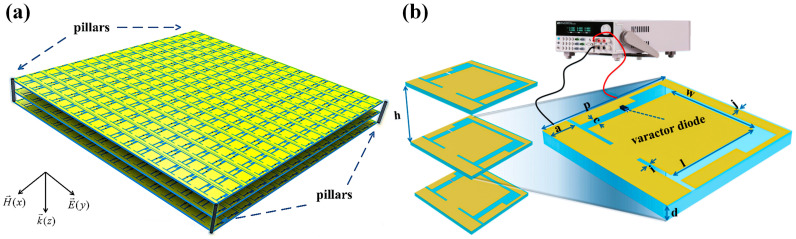
(**a**) Illustration of the active transmissive metasurface part; (**b**) schematic diagram of the unit cell, and the inset shows the dimensions of the unit cell.

To realize the information modulation, we design the active transmissive metasurface part with the phase-shifting capacity. The schematic of the proposed metasurface is shown in Figure 1a. It is a triple-layered structure containing an array of 16 × 16 unit cells in each layer. The three layers are identical in geometries and separated by air gaps of the same height. There are four small nylon pillars at the corners to fix the whole structure. From Figure 1b, the unit pattern looks like a complementary ring structure standing on top of the substrate F4B ($\epsilon_{r}$ = 2.2 and tanδ = 0.0009). A varactor diode is used to connect the outer ring and internal patch, in order to provide a variable capacitance that dependents on the applied voltage. The substrate has a thickness of *d* = 0.762 mm. The perimeter slot enhances structural capacitance and prevents DC short circuits between the metallic areas bridged by the varactor. The distances between adjacent substrates are *h* = 10 mm. Other parameters shown in Figure 1b are *a* = 2.5 mm, *c* = 1 mm, *i* = 0.4 mm, *j* = 0.2 mm, *l* = 10 mm, *w* = 10 mm, and *p* = 15 mm.

From the classical EM theory, the proposed metasurface can be regarded as a spatial band-pass filter when interacting with EM waves. An equivalent circuit model can be employed to describe the spectral properties of the unit cell. At the resonant frequency, the electrons on the metallic patterns absorb the majority of the incident energy and then radiate it outwards through the slot. As a result, the metasurface has a high transmission efficiency in a narrow passband around the resonant frequency. The varactor diode, named SMV1405 (Skyworks Ltd., Shanghai, China), can be modeled as a resistor–capacitor–inductor (*R*-*C*-*L*) equivalent circuit with the values of *R*, *L*, and *C* of varactor under different biasing voltages given in Table 1. The operating mechanism of the phase-shifting metasurface can be described as follows: each unit cell acts as a complementary ring resonator, and a varactor diode bridging the outer ring and internal patch. When we change the applying bias voltage, the junction capacitance of varactor can be altered; such capacitance shift can modify the resonant frequency of unit cell, thereby changing the transmission phase.

**Figure 2:**
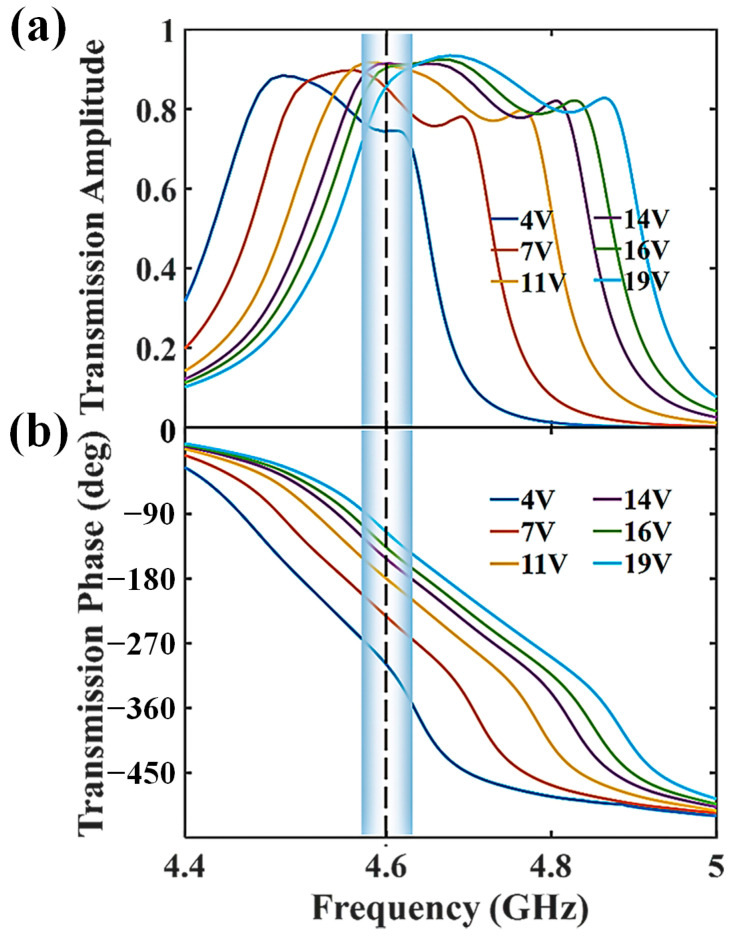
(**a**,**b**) Simulated transmission amplitude and phase spectra of the unit cell under different bias voltages. The highlighted region stands for the operation bandwidth.

To investigate the performance of the metasurface, the unit cell is simulated via a commercial electromagnetic solver (CST Microwave Studio 2022, Shanghai Ruambo Engineering Software Co., Ltd., Shanghai, China), when the bias voltage is gradually increased from 4 V to 19 V. During the simulation, the electrical and magnetic walls are applied along *x-* and *y*-axes in Figure 1a to mimic a two-dimensional infinite surface. A plane wave is used to illuminate the metasurface normally as the excitation. Figure 2a,b demonstrate the transmission amplitude and phase of the unit cell as a function of the biasing voltage. It can be seen that the realized transmission amplitude is always higher than 0.7 within the bandwidth of interest. At the same time, a phase range of 180° can also be achieved when the applied voltage is altered. The region that fits for BPSK baseband modulation is highlighted in Figure 2, with the operating frequency marked by a vertical dashed line. It is clear that the unit cell can operate well within the frequency range of 4.6 ± 0.03 GHz—that is to say, the total bandwidth is nearly 60 MHz. As shown in Figure 2, after evaluating the transmission amplitude and phase difference together, we choose the ‘0’ and ‘1’ elements under the bias voltages of 4 V and 19 V, respectively. The corresponding transmission coefficients are called as $T_{0}$ and $T_{1}$. Since all the unit cells have identical patterns, a uniform transmission response can be obtained across the surface.

**Figure 3:**
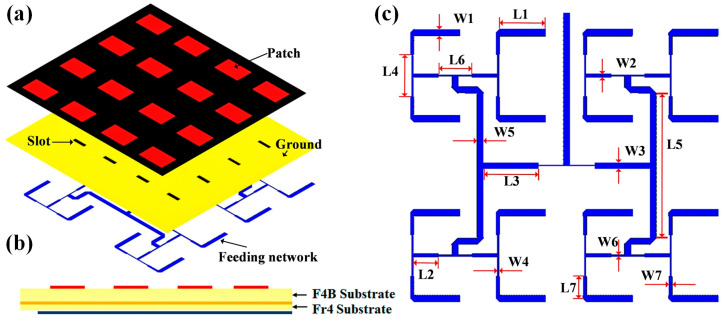
Layout of the antenna array as the feeding source of the metasurface. (**a**) Exploded 3D view; (**b**) side view; (**c**) bottom view. The detailed dimensions are $L_{1}$ = 21.75 mm, $W_{1}$ = 3 mm, $L_{2}$ = 12 mm, $W_{2}$ = 1.6 mm, $L_{3}$ = 26 mm, $W_{3}$ = 3 mm, $L_{4}$ = 20 mm, $W_{4}$ = 0.65 mm, $L_{5}$ = 69 mm, $W_{5}$ = 3 mm, $L_{6}$ = 16 mm, $W_{6}$ = 0.65 mm, $L_{7}$ = 10.4 mm, $W_{7}$ = 1.6 mm.

To construct the radiation-type metasurface structure, we design a small antenna array of 4 × 4 microstrip patches, which air-gaped from the transmissive metasurface to feed the metasurface with a low profile, as demonstrated in Figure 3. The patch array is located on a F4B dielectric substrate with the thickness of 4 mm. Its feeding network lies at the bottom of another substrate (FR4, $\epsilon_{r}$ = 4.4, thickness: 1.2 mm) below the F4B layer. A copper film is inserted between the two substrates as the ground, with rectangular slots periodically etched below the patches. Microwave signals from the feeding network can be efficiently conveyed to the patches through the slot coupling. The dimensions of the metallic rectangular patch and the slot are 16.5 mm × 24.5 mm and 3.5 mm × 15 mm, respectively. A T-shaped power divider is employed to feed the antenna elements with the equal amplitudes and phases.

**Figure 4:**
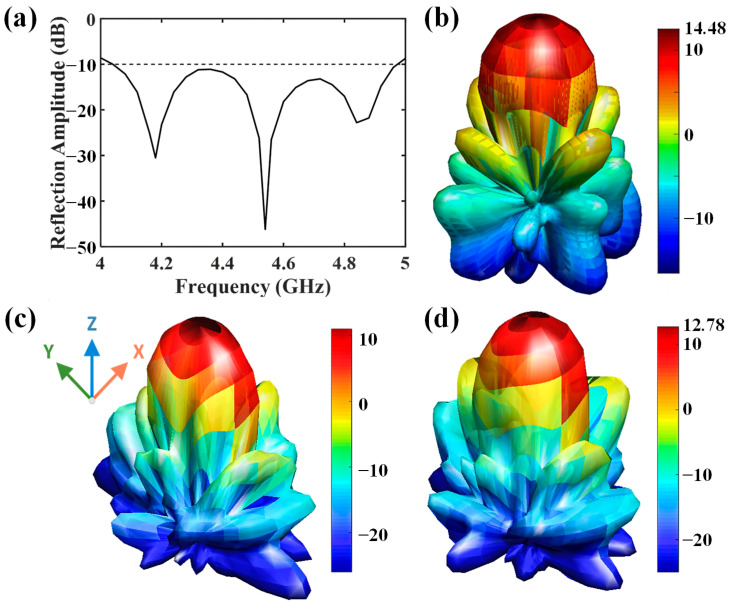
(**a**) Reflection coefficient of the antenna array; (**b**) simulated radiation pattern of the antenna array at 4.6 GHz; (**c**,**d**) simulated radiation pattern of the antenna array with the metasurface cover at 4.6 GHz, where the unit cells work in ‘0’ and ‘1’ states, respectively.

Figure 4a gives the reflection coefficient of the proposed antenna array. It shows an impedance bandwidth from 4.04 GHz to 4.96 GHz with the reflection coefficient below −10 dB, which can cover the required operation frequency range of the metasurface. As shown in Figure 4b, the antenna array exhibits a good directive radiation pattern. The maximum gain reaches 14.48 dB at 4.6 GHz. When covered by the metasurface, the antenna gain is reduced because of the transmission loss from the elements, as illustrated in Figure 4c,d. Note that when the working state of the element is changed from ‘0’ to ‘1’, the antenna gain is increased from 11.04 dB to 12.78 dB, since in the two cases the element transmission losses are different from Figure 2. But the impact of the gain inconsistency can be neglected during the demodulation process, because only the transmitted phases are concerned in the recovery of the baseband information.

**Figure 5:**
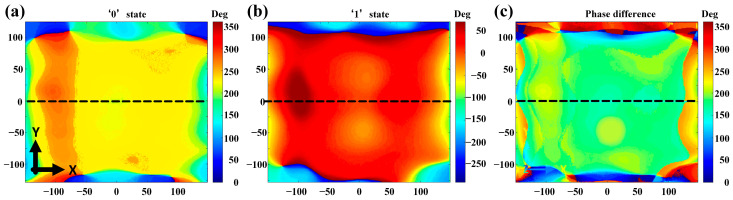
(**a**,**b**) Phase distributions of the metasurface under the excitation of the array antenna at 4.6 GHz, where the unit cells work in ‘0’ and ‘1’ states, respectively; (**c**) phase difference distribution between the two states.

Another important factor to study is the near-field phase distributions of the whole structure. As mentioned above, in the numerical simulations we assume normal incidence of plane waves upon the metasurface. However, due to the limited distance between the metasurface and the feeding antenna, the former is actually located in the near-field region of the latter part. As a result, it is necessary to check the transmitted field uniformity under the excitation of the antenna array. In Figure 5a,b, the phase distributions at the metasurface are monitored when the elements are in ‘0’ and ‘1’ states, we can find that there are large phase distortions near the structural edges. But fortunately, the phase differences of the transmitted electric field in both states are nearly constant. The phase difference is kept near 180 degrees as desired (Figure 5c), although inevitable phase fluctuations do exist across the whole metasurface under the near field excitation. Consequently, the radiation-type metasurface, enabling radiation and phase modulation functions, can realize the direct radiation-wave modulation and wireless communication.

**Figure 6:**
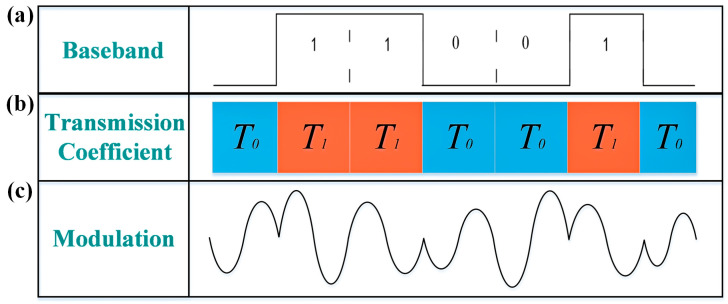
(**a**) Baseband information; (**b**) corresponding transmission coefficient sequences; (**c**) modulated signal waveforms.

To realize the wireless communication, a mapping relationship must be established. Taking BPSK modulation as an example, the two constellation points with opposite phases correspond to the transmission coefficient of $T_{0}$ and $T_{1}$, which can be encoded as ‘0’ and ‘1’, respectively. For instance, if the baseband data is ‘0110010’, the transmission coefficients of metasurface should be rapidly switched with a sequence of ‘$T_{0}T_{1}T_{1}T_{0}T_{0}T_{1}T_{0}$’. This switching process can be efficiently implemented using a field programmable gate array (FPGA) that controls the biasing voltages to drive the metasurface. The entire modulation process is illustrated in Figure 6. When the transmission phase alternates between $T_{0}$ and $T_{1}$, the baseband information can be directly loaded into the carrier wave and radiated into the free space, as shown in Figure 6a,b. The discontinuities observed in the modulated waveform result from the abrupt phase jumps between the two states. The modulated signal waveforms are provided in Figure 6c.

## 3. Measurement Results and System Demonstration

**Figure 7:**
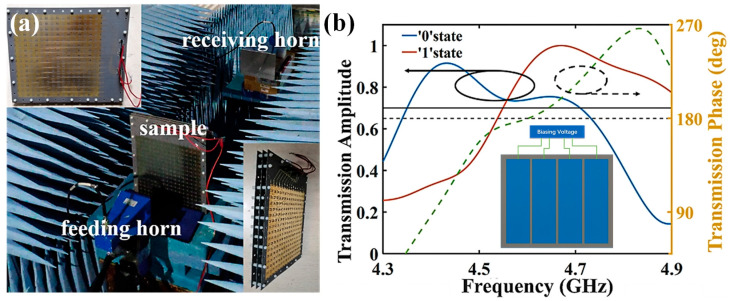
(**a**) Experimental configuration of the metasurface measurement; (**b**) measured transmission amplitude of the fabricated sample under ‘0’ and ‘1’ states, and the corresponding phase difference from 4.3 GHz to 4.9 GHz. The inset shows the schematic of the metasurface in which four adjacent columns share the same biasing voltage.

To validate the feasibility of above-mentioned design, the transmissive metasurface sample was fabricated based on standard printed circuit board (PCB) technology and measured in a microwave anechoic chamber. Figure 7a shows the sample and experimental configuration photograph. The sample has an overall size of 276 mm × 330 mm. In the experiment, every four adjacent columns share the same control voltage from a DC source, as displayed in the inset of Figure 7b. Two horns are used in the measurement. They were connected to the two ports of the vector network analyzer (VNA, Ceyear 3672D, Ceyear Technologies Co., Ltd., Qingdao, China) for transmitting and receiving, respectively. A DC source was connected to sample to provide external DC biasing voltages, and the sample was placed between the two antennas. The centers of the horns and the sample are adjusted to the same height to ensure the normal incidence. We surrounded the sample with microwave absorbers to minimize environmental reflections.

**Figure 8:**
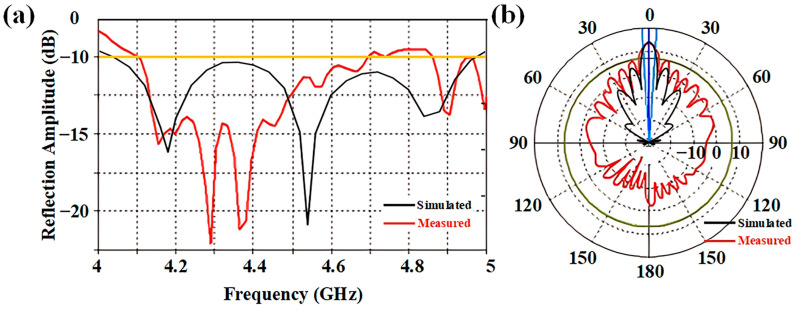
(**a**) Simulated and measured reflection coefficient of antenna prototype; (**b**) simulated and measured radiation pattern of the antenna prototype at 4.6 GHz.

In the experiment, we initially start to find out the transmission characteristics of the metasurface. The observation bandwidth of the VNA is set from 3 GHz to 6 GHz. The biasing voltage is changed to monitor the variance of transmission phase. As shown in Figure 7b, we give the transmission phases of ‘0’ and ‘1’ units, when the applied voltages are 4 V and 19 V, respectively. The phase difference between the two states is also provided for better illustration. It can be seen that the measurement is in good accordance with the simulation. A phase difference of 180 degrees can be obtained near 4.6 GHz as expected, which is sufficient to meet the phase demand of BPSK modulation. Good transmission amplitudes for the two units are also found in Figure 7b, implying moderate material loss during data transmission. Consequently, we conducted the performance measurement of proposed antenna array. Figure 8 shows the measured reflection coefficient and radiation pattern of the antenna prototype at 4.6 GHz. We found that although the measured results deviated from the simulation, it exhibited good radiation performance near 4.6 GHz, confirming the practicality of proposed radiation-type metasurface.

Then, we proceed to investigate the performance of the metasurface-based BPSK wireless communication system. The communication process can be described as follows: Firstly, the computer converts the message to be sent into binary bit streams and sends them to FPGA module, generating a ‘0’ and ‘1’ control sequence, which is output by a digital–analog conversion (DAC) module next. After translating the bit streams into the biasing voltage sequences, the metasurface turns to alter the phase of the transmitted waves from the feeding antenna in a predesigned fashion. The transmitted signals are finally detected by the receiving antenna after transmission over air and demodulated at the receiver end to restore the baseband data.

**Figure 9:**
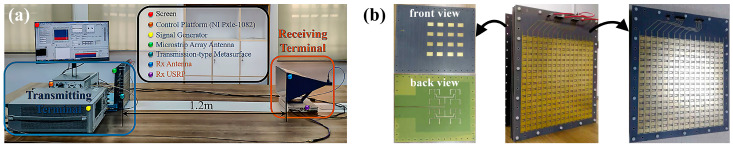
(**a**) Experimental configuration for metasurface-based BPSK system; (**b**) photographs of microstrip antenna, transmissive metasurface, and radiation-type metasurface.

The hardware architecture of the above-mentioned system is illustrated in Figure 9a, and Figure 9b illustrates the photographs of microstrip antenna, transmissive metasurface, and radiation-type metasurface. During the experiment, the radiation-type metasurface operates at 4.6 GHz, which is fed by the signal generator (Agilent E8267D, Agilent Technologies, Santa Rosa, CA, USA). The working states of the metasurface are controlled by an FPGA module, a DAC module, and a high-speed I/O bus controller, enabling real-time information bit conversion to biasing voltages instantly and rapidly. Meanwhile, the software-defined radio (SDR) reconfigurable device (USRP-2943, National Instruments, Austin, TX, USA) is utilized to demodulate signals from the receiving antenna. The distance between the transmitter and the receiver is 1.2 m. In the measurement, all the instruments are synchronized through phase-stable cables that connect the transmitting and receiving terminals. Additionally, some conventional communication technologies like cyclic prefix and cyclic redundancy check are also applied in this system to improve reliability and reduce the error rate during data transmission.

**Figure 10:**
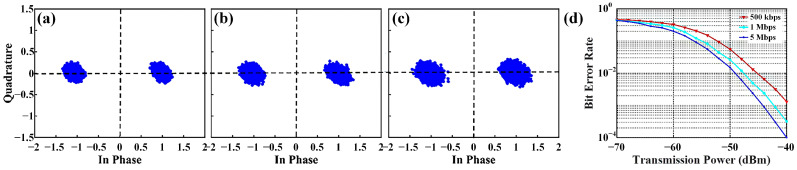
(**a**–**c**) Measured constellation diagrams under the transmission rate of 500 kbps, 1 Mbps, 5 Mbps, respectively; (**d**) relationship between the measured bit error rate and the transmitting power under different transmission rates.

Figure 9a illustrates the wireless video transmission scene, and the data transmission rate can reach 5 Mbps. A series of indoor tests are carried out based on above BPSK communication system. The measured constellation diagrams under different transmission rates are displayed in Figure 10a–c. The two constellation points exhibit a 180° phase difference and stand for the binary symbols ‘0’ and ‘1’, respectively. With the growth of the transmission rate, error vector magnitude (EVM) becomes larger owing to the increased noise during transmission, but the system maintains robust data transmission, validating the practicality of the proposed communication scheme. The dependence of the bit error rate (BER) on the transmission power is illustrated in Figure 10d. It is worth noting that the BER increases as the transmission rate goes up. This is reasonable since a faster transmission rate results in wider signal bandwidth. Under the same transmission power, wider signal bandwidth leads to higher noise power and increases the probability of misjudgments.

## 4. Conclusions

In this work, we propose a radiation-type digital coding metasurface for wireless communication distinct from the reported reflection-type metasurface. The new metasurface offers structural simplicity, a low profile, and easy integration. By incorporating varactor diodes into the unit cells, the transmission phase of the metasurface achieves binary phase switching (0° and 180°), enabling 1-bit phase quantization at the surface. A microstrip array antenna serves as the feeding source to provide quasi-plane wave excitation. Based on the proposed metasurface, we established a BPSK wireless communication system and measured its performance. The direct signal modulation approach simplifies the architecture of traditional communication system and demonstrates strong potential for 6G applications.

By key metric comparisons between our work and the cited references (Refs. [23,24]), the novelty of the proposed radiation-type metasurface is highlighted in Table 2:

In conclusion, the advantages of our work are high system integration, real-time signal modulation, and practical communication performance, which contribute to establishing a new paradigm for metasurface-based compact transceivers in 6G networks.

## Figures and Tables

**Table 1:** Equivalent capacitance, resistance, and inductance of the varactor diode (SMV1405) as a function of the biasing voltage.

Bias Voltage (V)	Varactor Capacitance (pF)	Varactor Resistance (Ω)	Varactor Inductance (nH)
4	1.24	0.47	0.7
19	0.76	0.31	0.7

**Table 2:** Key metric comparisons between our work and the cited references.

Metric	Ref. [23]	Ref. [24]	Our Work
Operational bandwidth	Not explicitly quantified	~40 MHz	60 MHz (at 4.6 GHz)
Modulation speed	Not demonstrated	Not demonstrated	5 Mbps
System integration	Single-layer metasurface	Dual-function control	Antenna-integrated metasurface
Application focus	Generalized EM manipulation	Reflection/transmission control	Wireless communication
Experimental validation	Far-field beam steering	Scattering control	Real-time signal transmission

## Data Availability

Data is contained within the article; further inquiries can be directed to the corresponding author.
